# The Revised Uniform System of Accounts

**Published:** 1915-08-28

**Authors:** 


### THE REVISED UNIFORM SYSTEM OF ACCOUNTS.

### The Three Funds Satisfied.

Between now and the end of the year the prudent hospital secretary or accountant will look carefully through what is colloquially known as "The Red Book" and satisfy himself that the requirements of the three Funds are duly being carried out in the books under his care.

The Uniform System was never as the laws of the Medes and Persians. Even if it were not desirable to review from time to time hospital accountancy in the light of ripened experience, the passing of new legislation, such as the National Health Act, would necessarily bring changed methods and demand amended forms of account. The desire that every hospital administrator has to fall in with, and conform to the spirit of, the directions of the Funds, and to carry them out exactly in the same way as he expects others to observe them, will cause him to read attentively those paragraphs in the Red Book which contain either new regulations or explanatory additions to existing regulations. This exercise will not preclude him from considered criticism, for he knows that it is chiefly due to the cumulative effect of such criticism that the chief recent reforms in hospital accountancy have been brought about.

It may be found helpful if we review in their paragraph order the directions which refer to complete alterations in practice or which qualify previous rulings.

### Section I.—Income and Expenditure Account.

*Paragraph 10.*—For the first time it is required that the cost of raising money through agencies specifically associated with a hospital shall be shown as expenditure, and the gross amount collected is to be placed on the income side. This logical arrangement has been followed by most hospitals, but it has, no doubt, been found desirable to make the rule for the guidance of the minority.

*Paragraph 11.*—Here the value of gifts in kind is directed to be introduced on both sides of the income and expenditure account. The paragraph, not being sufficiently explanatory, has been amplified by a special circular, and the whole matter has been dealt with in these columns in a recently published article on the King's Fund Statistics.

*Paragraph 15.*—Every annual subscription and donation received after midnight of December 31 must be included in the income for the new year. This is to say that a secretary, when he is paid a subscription for 1915 on January 1, 1916, no matter what was the cause of the delay, and is banking on that date amounts received after bank hours on the previous evening as well as the amounts received on New Year's Day, must not include the belated subscription in the 1915 "pay-in." In view of the well-known and praiseworthy eagerness of every secretary to keep up his annual subscription total, and with regard to the possibility of offending an annual subscriber by showing him as in default, it must be said that almost superhuman fidelity to a system is required in the case quoted.

*Paragraph 16.*—Contributions to make up deficits of income must not be excluded from the income and expenditure account. Contributions to reduce debt on building or capital account go straight to the balance sheet.

### Entertainments and Dinners.

*Paragraph* 17.—The gross receipts of festivals, bazaars, or other entertainments for the general purposes of a hospital, and the gross proceeds of the sale of dinner tickets for the same object, are to be entered as ordinary income under the heading of "Entertainments." The expenses are to be entered as ordinary expenditure under the heading "Administration," "Finance: 3. Festivals, Bazaars, etc." Annual subscriptions and donations received or promised at a dinner are to be placed under the ordinary headings. As is well known, the tickets for a festival dinner are generally free; the right kind of people are invited to the dinner, and it is hoped that whether they accept or not they will send a contribution to the funds. The dinner itself (apart from such contributions) always shows a loss, and where no tickets are sold, if the above directions are literally carried out, there would be substantial expenditure on the one side, and no visible income on the other. This would never do, and the way to get over the difficulty is to separate the receipts under subscriptions and donations between Annual Subscriptions (or Donations) and Annual Subscriptions (or Donations)—Festival Dinner. There have been no dinners this year, so the question will not arise, but no doubt the next issue of the Red Book will put right an obvious error.

*Paragraph* 18.—Uniformity is secured as to the allocation of dividends or interest from investments.

### Receipts under the Insurance Act.

*Paragraph* 21.—The directions have reference to sums received in respect of patients. In a few words, sums received as the result of an arrangement with an Insurance Authority, Approved Society, or other Agency under the Act must be placed under the new heading. Voluntary payments by insured patients must be included under "Patients' Payments." As was pointed out in a recent article in The Hospital, on the payment by insured in-patients without dependants of sickness benefit to the hospital they enter, there is more than one method of agreement, and to secure complete uniformity this point must be studied.

### Washing.

*Paragraph* 23.—Arrangements are here made for the division under the proper headings of the cost of washing done on the premises. No method has yet been devised of indicating in the income and expenditure account the cost of provisions consumed by the laundry staff, but in the washing return (printed separately in the annual reports) there is a line for this expenditure.

Renewals and Repairs.

*Paragraph* 33.—We quote the words that are the essence of the paragraph: "Expenditure which is necessary for the ordinary upkeep and working of an institution does not become 'extraordinary' because it happens to be exceptional in amount in a particular year—whether exceptionally large or exceptionally small."

Section II.—The Balance Sheet.

Most of the paragraphs amplify or make previous published directions clearer. We, therefore, call attention to those only that are of special importance. In dealing with the capital account, stress is laid upon the definition of an endowment as regards the balance sheet itself. Endowment is used throughout the Revised Uniform System to signify a fund which is subject to a special trust, attached by the donor, for the retention of the capital by the hospital. Hospital endowments, then, as we are expected to use the term, have nothing to do with investments of surplus income.

Investments: Valuation and Depreciation.

*Paragraph* 13.—Investments should be stated at cost prices, except in cases where important depreciation believed to be of a permanent character has taken place, when the cost price may be written down and the security stated at the lower value. The basis of valuation should be stated thus: "Value at cost," "Valued at date of receipt," "Valued at December 31st, 191-," etc., as the case may be.

We presume the obvious advantage of having a uniform date for valuation has to be put on one side, because of the difficulty and cost of obtaining a valuation of that part of a hospital's investments which are represented by real estate.

Transfer to Capital of Income Moneys Invested.

*Paragraph* 22.—Instead of temporary investments appearing under a special heading as heretofore, they should, if intended to be more or less permanently invested, be transferred to Capital Account "(C) For General Purposes." Otherwise no transfer should be made, and the Capital Account for General Purposes will appear for the time being as over-invested.

Other paragraphs containing minor alterations or qualifications should be carefully studied, but on the whole the balance sheet is altered only by the provisions of Paragraph 22, and the instructions in respect of the basis of valuation of investments.

Definition of an Out-Patient.

The following definition of an out-patient is worth recording: for statistical purposes an out-patient is a patient attending continuously for the same ailment, for however long a period, between January 1 and December 31 inclusive. A casualty patient is either an in-patient or an out-patient. If taken in, he counts as an in-patient. If treated and sent home, he counts as an out-patient. Where casualties are distinguished from out-patients, care must be taken that no patient is counted twice. An ordinary out-patient admitted to the wards, whether on the first or any subsequent visit, should be counted both as an out-patient and as an in-patient.

*Audit of Accounts*.—It is requested that the chartered or incorporated accountant who undertakes the audit of the accounts shall satisfy himself that all the figures are prepared and stated in accordance with the Revised Uniform System.

